# Modified Huangqi Chifeng decoction alleviates IgA nephropathy inflammation and renal fibrosis via miR-146a/TLR4/NF-κB signaling modulation

**DOI:** 10.3389/fimmu.2026.1867439

**Published:** 2026-07-08

**Authors:** Yue Shi, Jing Liu, Sijia Ma, Hangyu Duan, Xiujie Shi, Meiying Chang, Bin Yang, Mingming Zhao, Yu Zhang

**Affiliations:** 1 Department of Nephrology, Xiyuan Hospital, China Academy of Chinese Medical Sciences, Beijing, China; 2 Integrated Traditional Chinese and Western Medicine Diagnosis and Treatment Center, Chongqing Academy of Medical Sciences, Chongqing General Hospital, Chongqing University, Chongqing, China; 3 Beijing University of Chinese Medicine, Beijing, China; 4 The Third Affiliated Hospital of Zhejiang Chinese Medical University (Zhongshan Hospital of Zhejiang Province), Hangzhou, China; 5 Deparment of Nephrology, The First Hospital of Tsinghua University, Beijing, China; 6 Department of Pathology, Xiyuan Hospital, China Academy of Chinese Medical Sciences, Beijing, China

**Keywords:** IgAN, immunoglobulin A nephropathy, inflammation, miR-146a, modified Huangqi Chifeng decoction, renal fibrosis, TLR4/NF-kB

## Abstract

**Background:**

Immunoglobulin A nephropathy (IgAN) is the most prevalent primary glomerulonephritis worldwide and a leading cause of end-stage renal disease, posing a significant threat to human health. Modified Huangqi Chifeng decoction (MHCD) has shown efficacy in ameliorating IgAN; however, its underlying regulatory mechanisms remain incompletely understood.

**Objective:**

To evaluate the anti-inflammatory and anti-fibrotic effects of MHCD on IgAN and elucidate its molecular mechanisms through *in vivo* and *in vitro* experiments.

**Methods:**

An IgAN rat model was established. The therapeutic effects of MHCD were assessed by measuring 24-hour urinary protein, Gd-IgA1, serum biochemistry, renal pathological damage, IgA deposition, inflammatory mediators, and fibrosis levels after 8 weeks of MHCD administration. ELISA was used to quantify inflammatory mediators; Western blot and immunohistochemistry were employed to analyze protein expression; PCR was performed to determine miR-146a levels. Subsequently, lipopolysaccharide-stimulated mesangial cells were used *in vitro*. Mesangial cell proliferation and the expression of TLR4/NF-κB signaling pathway components and downstream inflammatory factors was examined to verify the anti-inflammatory and anti-fibrotic mechanism of MHCD by overexpressing or silencing miR-146a.

**Results:**

MHCD reduced urinary protein and serum Gd-IgA1 in IgAN rats, and alleviated renal IgA deposition, pathological injury, inflammation and fibrosis. Furthermore, MHCD inhibited renal expression of TLR4, MyD88, NF-κB p65 and NF-κB p-p65, and increased the level of miR-146a. Mechanistically, *in vitro* experiments demonstrated that MHCD exerted anti-inflammatory and anti-fibrotic effects by upregulating miR-146a and inhibiting the TLR4/NF-κB signaling pathway.

**Conclusion:**

MHCD alleviates IgA nephropathy inflammation and renal fibrosis partially through upregulating miR-146a and subsequently suppressing the TLR4/NF-κB signaling pathway.

## 1 Introduction

Immunoglobulin A nephropathy (IgAN) is the most common primary glomerulonephritis worldwide, with significant regional differences in its incidence. The incidence rate in Asia is much higher than that in Europe and America (1). It is reported that IgAN accounts for approximately 54.3% of all renal biopsy cases in China (2). IgAN is mainly characterized by the deposition of IgA or IgA-dominant immunoglobulins in the glomerular mesangial area. Its clinical manifestations are highly heterogeneous, ranging from mild hematuria or proteinuria to severe renal insufficiency. IgAN is an important cause of renal failure, with approximately 40% of patients progressing to end-stage renal disease (ESKD) within 10 to 20 years, posing a serious threat to patients’ lives and health (3).

The pathogenesis of IgAN is complex, with the currently widely accepted mechanism being the “four-hit” hypothesis. First, there is an overproduction of galactose-deficient IgA1 (Gd-IgA1). Next, autoantibodies targeting Gd-IgA1 are formed, leading to the creation of immune complexes. Subsequently, these immune complexes deposit in the glomerular mesangial area, triggering renal inflammation and mesangial cell proliferation (4, 5). Inflammation plays a critical role in the development of IgAN. A study integrating transcriptomic and proteomic analyses of renal biopsy specimens from IgAN patients revealed that Gd-IgA1 induces multiple pathways in mesangial cells that predominantly mediate inflammatory responses (6). Toll-like receptors (TLRs) are key components of the mammalian innate immune system. One of their primary signaling pathways involves the binding of myeloid differentiation factor 88 (MyD88) to the Toll/interleukin-1 receptor domain of TLRs, which activates the nuclear factor-κB (NF-κB) pathway, thereby promoting the secretion of numerous cytokines and influencing immune and inflammatory responses (7). Substantial evidence indicates that TLRs are involved in the production of Gd-IgA1. The kidneys of IgAN rat models exhibit upregulated TLR4 expression (8). Thus, TLRs and their signaling pathways contribute to renal inflammation and fibrosis in IgAN (9, 10). Blocking the TLR4/NF-κB signaling pathway-mediated immune-inflammatory response represents a potential therapeutic target for IgAN.

MicroRNAs are a conserved group of endogenous small RNA molecules that regulate the expression of target proteins through mRNA degradation or translational repression (11, 12). Among them, miR-146a is well-known for its immunosuppressive and anti-inflammatory effects. Studies have shown that miR-146a reduces IgA production in the gut, while miR-146a-deficient mice exhibit increased systemic IgA levels and enhanced renal IgA deposition, promoting the development of IgAN (13, 14). Extensive research has demonstrated that miR-146a suppresses the TLR4/NF-κB pathway, ameliorating various inflammatory diseases such as inflammatory bowel disease (15), chronic kidney disease (16), and hepatitis (17). However, the role of miR-146a in regulating the TLR4/NF-κB signaling pathway in IgAN has not yet been reported.

Recently, traditional Chinese medicine (TCM) has shown distinctive therapeutic benefits against inflammatory diseases. Accumulating evidence supports the extensive and effective use of TCM not only in kidney diseases but also in inflammatory disorders of other organs (8, 18–20). Thus, identifying effective TCM formulas and clarifying their anti-inflammatory mechanisms is of great significance for developing new treatments for inflammatory kidney diseases like IgAN. Professor Zhang Yu from Xiyuan Hospital, China Academy of Chinese Medical Sciences, based on his understanding of the pathogenesis of IgAN, developed an effective TCM compound—modified Huangqi Chifeng decoction (MHCD)—by refining and improving Huangqi Chifeng Tang, a classic formula from the renowned Qing Dynasty physician Wang Qingren, through decades of research. Our preliminary studies demonstrated that MHCD is safe and effective in reducing proteinuria in IgAN patients. Additionally, MHCD significantly inhibits lipopolysaccharide (LPS)-induced mesangial cell proliferation and extracellular matrix secretion, likely by suppressing the transforming growth factor-β1 (TGF-β1)/Smad signaling pathway (21). Further research revealed that MHCD attenuates renal inflammation in IgAN by downregulating the TLR4/NF-κB signaling pathway and the IL-17 signaling axis (22–24). However, the precise mechanisms underlying MHCD’s anti-inflammatory effects require further exploration. Therefore, this study combines *in vivo* and *in vitro* experiments to further investigate whether MHCD exerts its renal and mesangial cell protective effects via the miR-146a-mediated TLR4/NF-κB signaling pathway.

## 2 Materials and methods

### 2.1 Drugs and reagents

MHCD is composed of *Astragalus membranaceus* (Huangqi, 30g), *Paeonia lactiflora* (Chishao, 10g), *Saposhnikovia divaricata* (Fangfeng, 10g), *Dioscorea nipponica* (Chuanshanlong, 20g), *Euryale ferox* (Qianshi, 20g), *Rosa laevigata* (Jinyingzi, 10g), and *Hedyotis diffusa* (Baihuasheshecao, 20g). All the herbs are from the pharmacy of Xiyuan Hospital, China Academy of Chinese Medical Sciences (Beijing, China). After soaking for 30 minutes, the herbs were extracted twice with distilled water by boiling for one hour per cycle, using solvent-to-material ratios of 10:1 and 8:1 (v/w) for the first and second cycles, respectively. The combined decoctions were then filtered, concentrated under reduced pressure to a final concentration of 1.25 g crude drug/mL for the low dose and 2.50 g crude drug/mL for the high dose, and stored at −20 °C. Our previous study comprehensively analyzed the chemical profile of MHCD using ultra−performance liquid chromatography−tandem mass spectrometry (UPLC−MS/MS) and identified its major bioactive components, including paeoniflorin, 4′−O−β−glucopyranosyl−5−O−methylvisamminol, 5−O−methylvisamminol, sec−O−glucosylhamaudol, pseudoprotodioscin, calycosin, and formononetin (25).

MHCD-containing serum (MHCDS) was prepared. Thirty specific pathogen-free (SPF) male Sprague-Dawley (SD) rats (8 weeks old, weighing 180-220g) purchased from Sibeifu (Beijing) Biotechnology Co., Ltd. [SCXK (Jing) 2019-0010] were randomly divided into a control group (n=20) and an MHCDS group (n=10). Based on our prior experience, rats in the MHCDS group received 50g/kg MHCD (crude herb weight) by gavage once daily for 7 days, while controls received an equal volume of purified water (25). Two hours after the last administration, blood was collected from the abdominal aorta under anesthesia. The serum was separated by centrifugation at 3000 rpm for 10 min, heat-inactivated at 56 °C for 30 min, filtered for sterilization, and stored at -20 °C for further use.

The telmisartan tablets (Micardis) were obtained from Boehringer Ingelheim International GmbH (import drug registration number:H20171003). Bovine serum albumin (BSA), LPS, and carbon tetrachloride (CCl_4_) were procured from Sigma-Aldrich (St. Louis, MO, USA). Castor oil was sourced from Guangfu Fine Chemical Reagent Co., Ltd. (Tianjin, China).

Primary antibodies against fibronectin (ab268020), laminin (ab11575), TLR4 (ab22048), MyD88 (ab2064), NF-κB p65 (ab16502), and phospho-NF-κB p65 (Ser536; ab76302) were purchased from Abcam (Cambridge, UK).

Commercial ELISA kits for interleukin-4 (IL-4), monocyte chemoattractant protein-1 (MCP-1), and TGF-β1 were acquired from Sigma-Aldrich (St. Louis, MO, USA).

### 2.2 *In vivo* assays: IgAN rats

Fifty-six SPF male SD rats (8 weeks old, weighing 180–220 g) were supplied by Sibeifu (Beijing) Biotechnology Co., Ltd. [SCXK (Jing) 2019-0010] and housed in the SPF animal facility of Xiyuan Hospital, China Academy of Chinese Medical Sciences. The study protocol was approved by the Institutional Animal Care and Use Committee of Xiyuan Hospital (Approval No. 2022XLC028-2). After one week of acclimatization, all rats were randomly assigned to a control group (n = 13) and a model induction group (n = 43). The IgAN rat model was established following our previously described protocol. Briefly, rats in the model induction group received daily oral gavage of BSA at 600 mg/kg for 12 consecutive weeks, along with weekly subcutaneous injections of 0.3 mL castor oil mixed with 0.1 mL CCl_4_. Additionally, 0.05 mg LPS was administered via tail vein injection at weeks 8 and 10. Rats in the control group received equivalent volumes of normal saline following the same schedule. At week 13, 3 randomly selected rats from each group were sacrificed for model validation. The remaining 10 rats in the control group continued as the normal control. The 40 successfully modeled rats were then randomly divided into four groups: Model group (n=10), MHCD-L group (n=10, 12.5 g/kg based on crude herb weight), MHCD-H group (n=10, 25 g/kg based on crude herb weight), and positive drug Telmisartan group (n=10, 8.33 mg/kg). All treatments were administered once daily by oral gavage for 8 weeks, while control and model groups received equal volumes of purified water.

Twenty-four-hour urine samples were collected from all rats at 3-week intervals throughout the study. After 8 weeks of treatment, rats were anesthetized with intraperitoneal sodium pentobarbital (3%, 1.5 mL/100 g). Anesthesia was confirmed by loss of pedal reflex. Blood was collected from the abdominal aorta, after which rats were euthanized by exsanguination. Kidneys were harvested for further analysis. Serum biochemical parameters including serum creatinine (Scr), blood urea nitrogen (BUN), and albumin (ALB) levels were measured using standard laboratory methods. For histological examination, renal tissues were fixed in 4% paraformaldehyde solution. Additional kidney samples were immediately snap-frozen in liquid nitrogen and stored at -80 °C for future molecular analyses.

### 2.3 *In vitro* assays: LPS-induced mesangial cells

Mouse glomerular mesangial cells (MCs, GNM21) were obtained from the Cell Bank of the Committee for the Preservation of Typical Cultures, Chinese Academy of Sciences. The cells were cultured in Dulbecco modified Eagle medium (DMEM, Gibco, MD, USA) containing 10% fetal bovine serum, penicillin (100 U/mL) and streptomycin (100 μg/mL) at 37 °C and 5% CO_2_. MCs were treated with 10% MHCDS or 10% control serum plus LPS (10 ug/mL) for 48 hours (26). The MCs in the control group were not treated with LPS. The MCs involved in transfection were transfected first and then treated with LPS (10 ug/mL).

The miR-146a-5p inhibitor and mimic were commercially synthesized by Tsingke Biotechnology Co., Ltd (Beijing, China). For transfection experiments, MCs were transfected with either: miR-146a-5p inhibitor (50 nM), miR-146a-5p mimic (50 nM), and corresponding negative controls (50 nM). Using Lipofectamine™ RNAiMAX transfection reagent (Thermo Fisher Scientific, USA) according to the manufacturer’s protocol.

### 2.4 Renal histopathology

Renal tissue samples were fixed in 4% paraformaldehyde, dehydrated and paraffin-embedded, and then sectioned into 4 μm thin slices. The sections were stained with hematoxylin-Eosin (HE), Periodic Acid-Schiff’s stain (PAS) and Masson, and the pathological changes of glomeruli and renal tubules were observed through an optical microscope.

### 2.5 Immunofluorescence

Immunofluorescence assay was performed using renal tissue. The primary antibody was incubated at 4 °C overnight, followed by staining with a fluorescent secondary antibody. IgA deposition in the glomerular mesangial area was observed under a fluorescence microscope.

### 2.6 Immunohistochemistry

After deparaffinization and rehydration, the paraffin-embedded sections were subjected to heat-induced antigen retrieval in citrate buffer, followed by incubation with 3% hydrogen peroxide for blocking. The sections were then incubated with primary antibodies at 4 °C overnight. Subsequently, secondary antibodies were applied, and nuclei were counterstained with hematoxylin. After dehydration, clearing, and mounting, the sections were examined and imaged under a light microscope. The mean optical density (MOD) was used for quantitative analysis of fibronectin (FN) and laminin (LN) expression.

### 2.7 ELISA for IL-4, MCP-1, TGF-β1

The renal tissue was thoroughly homogenized and centrifuged to obtain the supernatant. The expressions of IL-4, MCP-1 and TGF-β1 in rat renal tissues were detected strictly in accordance with the instructions of the kit.

### 2.8 Western blot analysis

Total protein was extracted from renal tissues and cells using RIPA lysis buffer, followed by protein quantification via the BCA assay. The proteins were then separated by SDS-PAGE and transferred onto PVDF membranes. After blocking, the membranes were incubated overnight at 4 °C with the following primary antibodies: anti-TLR4 (1:1000), anti-MyD88 (1:1000), anti-NF-κB p65 (1:1000), and anti-NF-κB p65 (phospho S536, 1:1000). Subsequently, the membranes were probed with horseradish peroxidase-conjugated secondary antibodies at room temperature for 1 hour. Protein bands were visualized using enhanced chemiluminescence and analyzed with ImageJ software.

### 2.9 Quantitative real-time PCR

Total RNA was isolated using TRIzol reagent. Then, the total RNA was reverse transcribed and amplified by qPCR. The relative expression of the target gene was calculated by the 2^‐ΔΔCt^ method. The qPCR primer sequences are shown in **Table 1**.

**Table 1:** Sequence of primer pairs used for gene amplification.

Target gene	Forward primer	Reverse primer
TLR4	TTTATTCAGAGCCGTTGGT	GACAATGAAGATGATGCCAGA
MyD88	CTTCCAGACCAAGTTTGCACTCA	GCATATAGTGATGAACCGCAGGA
NF-KB p65	CGAGCTCAAGATCTGCCGAGT	CACAGCAAGAAGATCTCATCCCC
miR-146a-5p	TCGGCAGGTGAGAACTGAATTCCA	CTCAACTGGTGTCGTGGA
FN	GACCCCCTTCATCACCAACC	GGCCCGGAACATGAGGATAG
LN	CACTCCAAGCCCACAAAAGC	GGGCAGGAATGAGGGAAGAC
β-actin	CTCCTGAGCGCAAGTACTCT	TACTCCTGCTTGCTGATCCAC

### 2.10 CCK-8

MCs were seeded in 96-well plates. After treatment according to experimental groups, cells were incubated in a humidified atmosphere at 37 °C with 5% CO_2_ for 24 hours. Subsequently, 10 μL of CCK-8 solution was added to each well, followed by 2 hours of incubation. Absorbance was measured at a wavelength of 450 nm using a microplate reader.

### 2.11 Statistical analysis

Intergroup comparisons were performed using one-way analysis of variance, followed by Tukey’s *post hoc* test for pairwise comparisons. Statistical analyses were conducted using SPSS version 26.0 for Windows (SPSS Inc., Chicago, IL, USA). A *P*-value <0.05 was considered statistically significant.

## 3 Results

### 3.1 MHCD reduces proteinuria and serum Gd-IgA1 levels in IgAN rats

**Figure 1:**
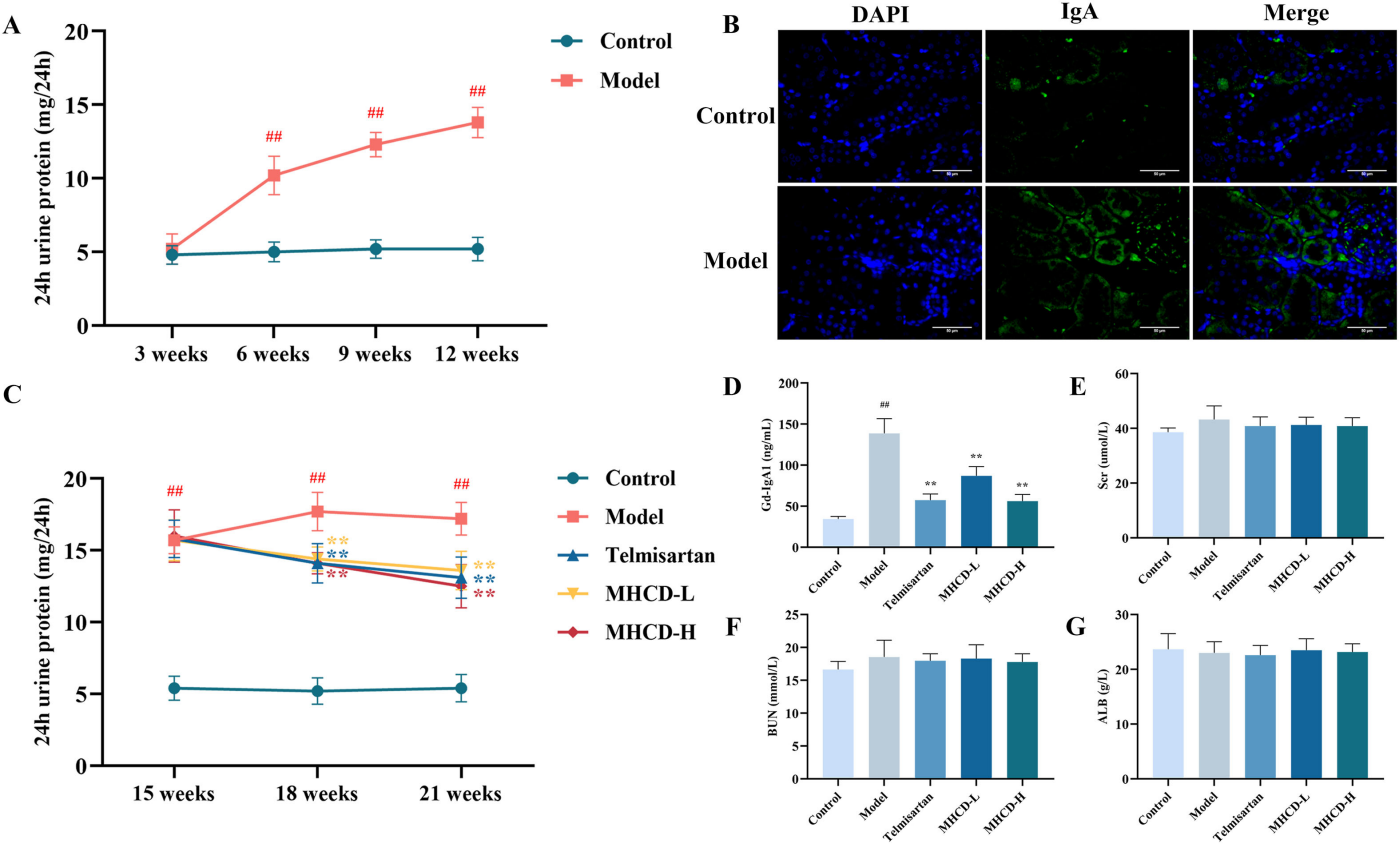
MHCD reduces proteinuria and serum Gd-IgA1 levels in IgAN rats. **(A)** 24-hour urinary protein during the 3–12 weeks (*n* = 10). **(B)** IgA deposition (*n* = 3; 400×; Scale bar: 50 μm). **(C)** 24-hour urinary protein during the 15–21 weeks (*n* = 10). **(D)** Gd-IgA1 results (*n* = 10). **(E)** Scr results (*n* = 10). **(F)** BUN results (*n* = 10). **(G)** ALB results (*n* = 10). ^##^*P <* 0.01 vs. control group; **P < 0.01 vs. model group.

As shown in **Figure 1A**, during the 6–12 weeks of modeling, the 24-hour urinary protein quantification in the model group rats was significantly higher than that in the control group. Immunofluorescence (**Figure 1B**) revealed pronounced IgA deposition in the glomerular mesangial area of the model group, confirming the successful establishment of the IgAN rat model.

Following successful model establishment, the rats were divided into groups and administered either high- or low-dose MHCD or telmisartan. As shown in **Figure 1C**, at the 18th and 21st weeks of the experiment, compared with the model group, the 24-hour urinary protein quantification was significantly reduced in the low-dose MHCD group, high-dose MHCD group, and telmisartan group (*P* < 0.01). After the intervention, serum levels of Gd-IgA1, SCr, BUN, and ALB were measured (**Figures 1D-G**). Compared with the model group, Gd-IgA1 levels were significantly lower in the low-dose MHCD, high-dose MHCD, and telmisartan groups (*P* < 0.01). However, there were no statistically significant differences in SCr, BUN, or ALB among the groups during the intervention period (*P* > 0.05).

### 3.2 MHCD ameliorates renal pathological injury in IgAN rats

**Figure 2:**
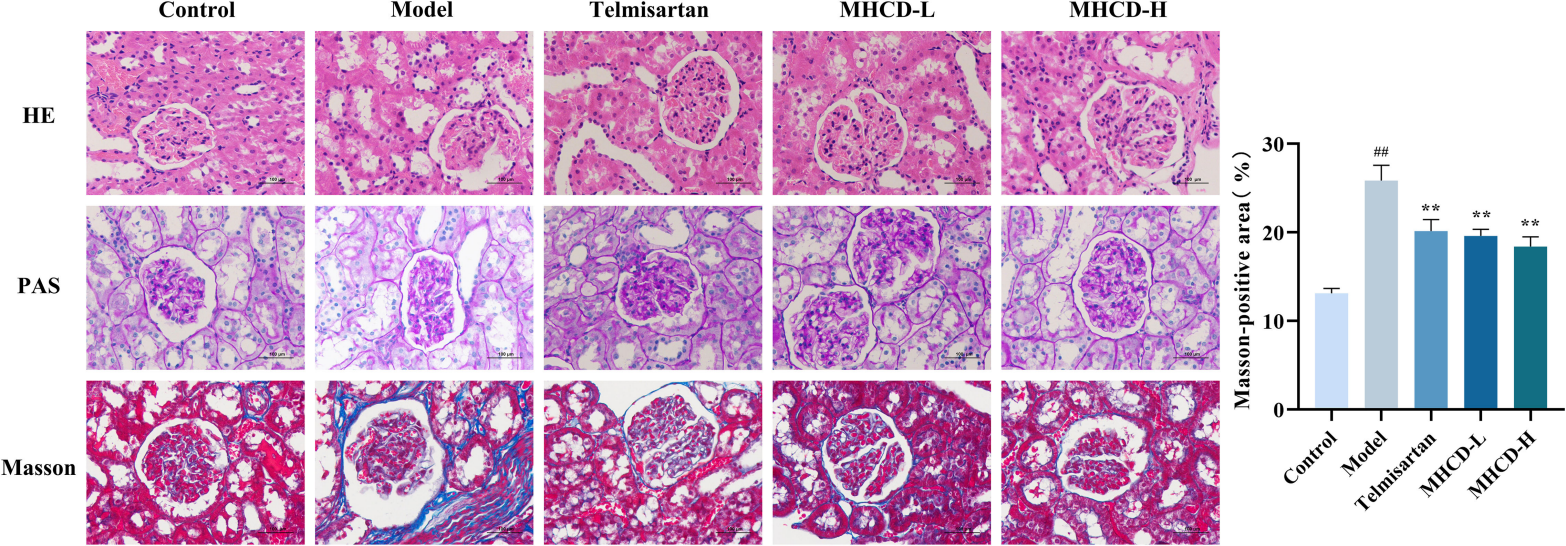
MHCD ameliorates renal pathological injury in IgAN rats (*n* = 6; 200×; Scale bar: 100 μm). ^##^P < 0.01 vs. control group; **P < 0.01 vs. model group.

Renal pathological changes were examined under light microscopy following HE, PAS, and Masson staining (**Figure 2**). In the control group, the glomeruli exhibited intact structures with clear vascular lumina, without significant mesangial hypercellularity or matrix deposition. Renal tubules were regularly arranged. In the model group, some glomeruli in the renal tissue presented lobulated or nodular structures, the balloon walls were uneven, the balloon Spaces were generally narrowed, the capillary endothelium proliferated, some glomerular capillaries were atretic or narrowed, inflammatory cell infiltration and interstitial fibrosis were relatively obvious, the basement membrane thickened, the arrangement of the lumen of the renal tubules was irregular, accompanied by compensatory atrophy or dilation, and vacuolar degeneration of epithelial cells. Both glomerular and tubular pathologies were ameliorated to varying degrees in the treatment groups (low-/high-dose MHCD and telmisartan).

### 3.3 MHCD reduces IgA deposition in the renal tissue of IgAN rats

**Figure 3:**
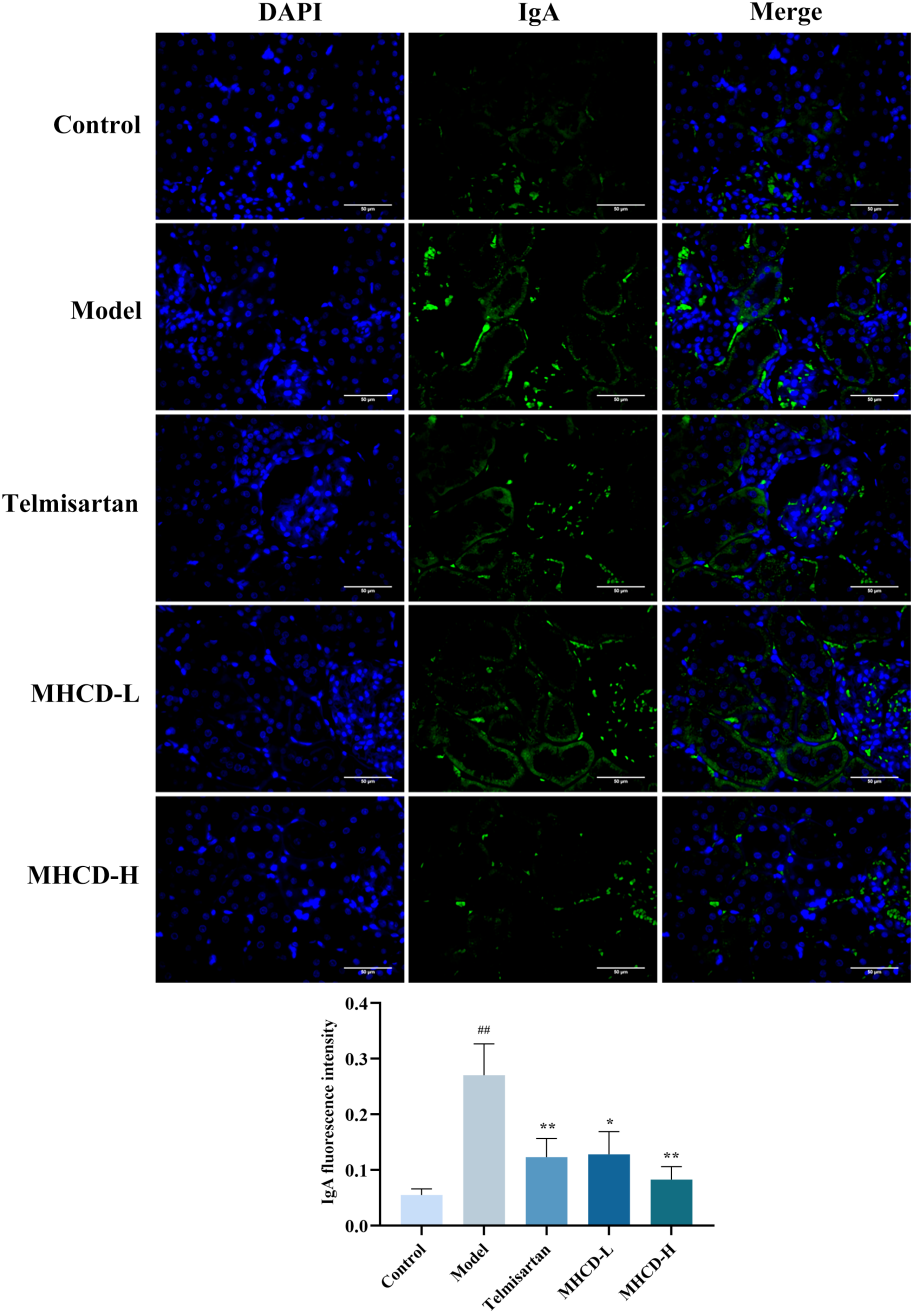
MHCD reduces IgA deposition in the renal tissue of IgAN rats (*n* = 3; 400×; Scale bar: 50 μm) ^##^*P <* 0.01 vs. control group; *P < 0.05 vs. model group; **P < 0.01 vs. model group.

The IgA deposition in rat renal tissue was observed by immunofluorescence (**Figure 3**). In control group specimens, glomerular mesangial areas exhibited negligible IgA immunofluorescence signals. Significant granular IgA deposition occurred in the glomerular mesangial matrix area of rats in the model group. The IgA deposition in the telmisartan group, the low-dose and high-dose MHCD groups all decreased to varying degrees (*P* < 0.05, *P* < 0.01).

### 3.4 MHCD alleviates renal fibrosis and inflammation in IgAN rats

**Figure 4:**
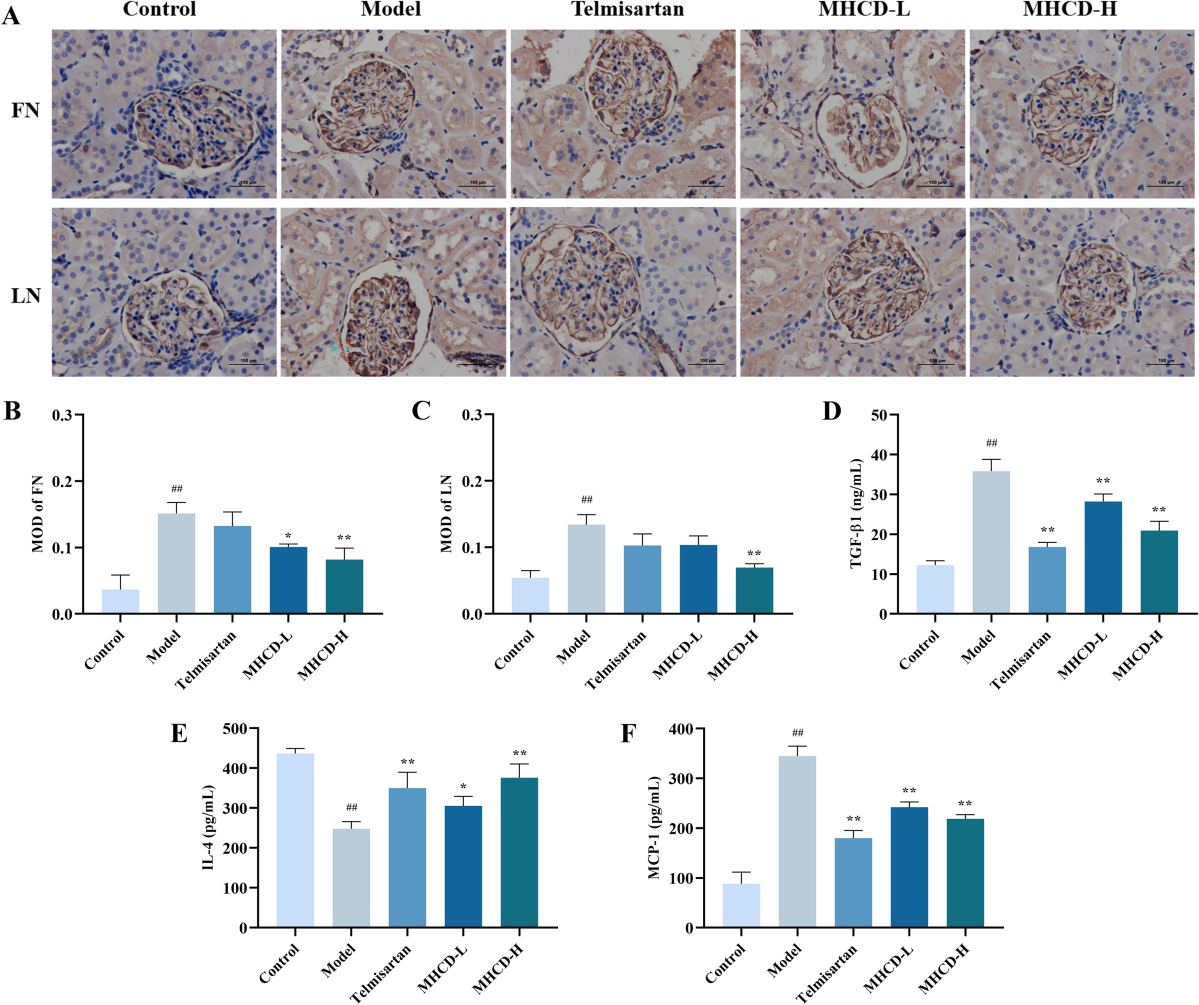
MHCD alleviates renal fibrosis and inflammation in IgAN rats (*n* = 3, 200×; Scale bar: 100 μm). **(A)** FN and LN immunohistochemical analysis. **(B)** MOD of FN. **(C)** MOD of LN. **(D)** TGF-β1 results. **(E)** IL-4 results. **(F)** MCP-1 results. ^##^*P <* 0.01 vs. control group; *P < 0.05 vs. model group; **P < 0.01 vs. model group.

Immunohistochemical analysis revealed the expression of FN and LN in renal tissues (**Figures 4A-C**). Compared with the control group, the expression levels of FN and LN in the renal tissues of the model group increased. High-dose MHCD significantly reduced the expressions of FN and LN in renal tissue (*P* < 0.01). ELISA quantification of inflammatory mediators (**Figures 4D-F**) showed marked upregulation of TGF-β1 and MCP-1 concomitant with IL-4 downregulation in model group kidneys (*P* < 0.01). Therapeutic intervention with either MHCD or telmisartan significantly reduced the expressions of TGF-β1 and MCP-1, and increased the expression of IL-4 (*P* < 0.05, *P* < 0.01), indicating that MHCD could alleviate renal fibrosis and inflammation in IgAN rats.

### 3.5 MHCD inhibits the TLR4/NF-κB signaling pathway in IgAN rats

**Figure 5:**
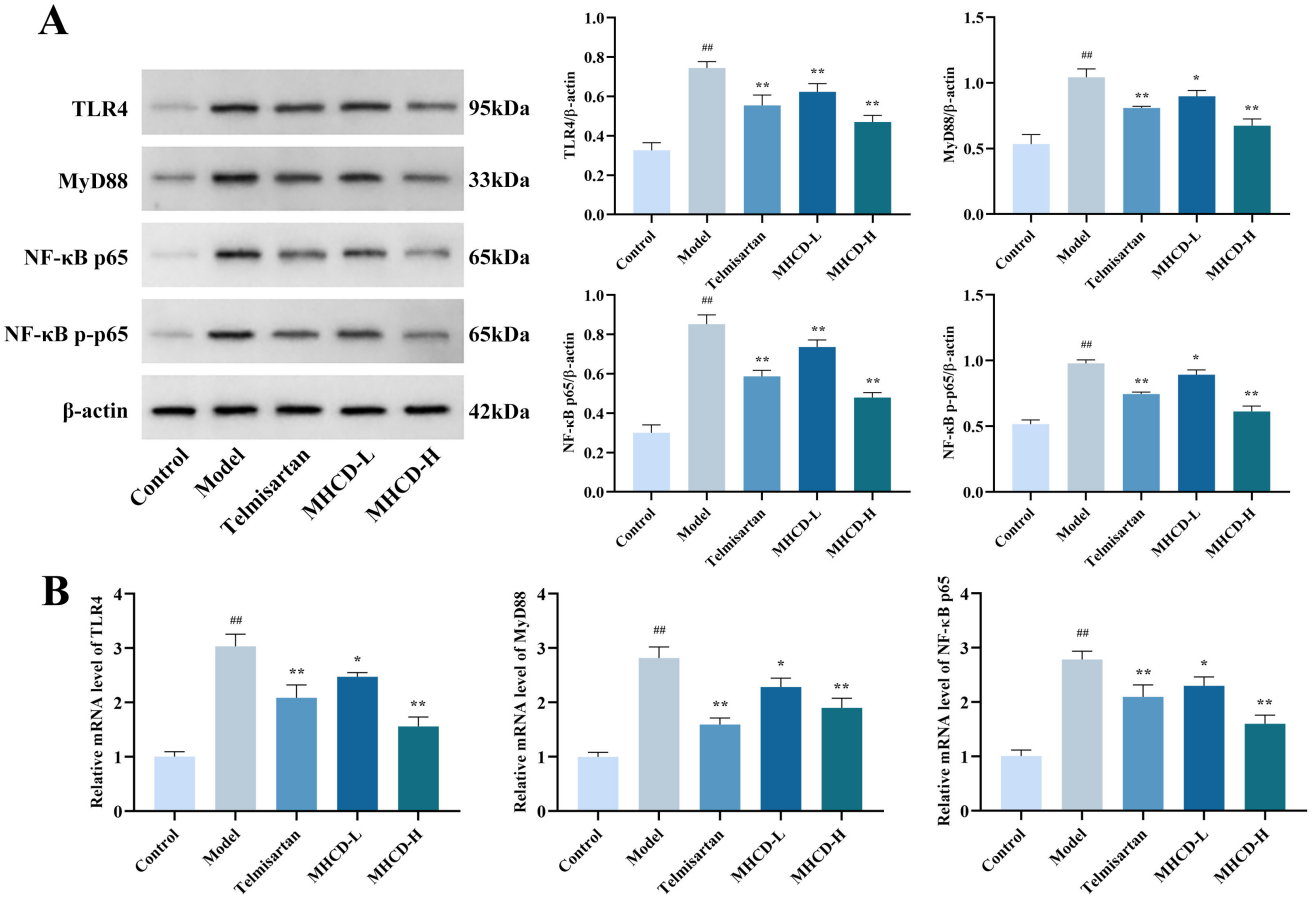
MHCD inhibits the TLR4/NF-κB signaling pathway in IgAN rats (*n* = 4). **(A)** Western blot analysis of TLR4, MyD88, NF-κB p65, and NF-κB p-p65. **(B)** Relative mRNA expression of TLR4, MyD88, NF-κB p65. ^##^*P <* 0.01 vs. control group; *P < 0.05 vs. model group; **P < 0.01 vs. model group.

Western blot analysis (**Figure 5A**) demonstrated significant upregulation of key signaling molecules in the model group, with markedly increased protein expression levels of TLR4, MyD88, NF-κB p65, and phosphorylated NF-κB p65 (NF-κB p-p65) compared to controls (*P* < 0.01). Therapeutic intervention with either MHCD or telmisartan resulted in dose-dependent attenuation of this signaling cascade, as evidenced by significantly reduced expression of TLR4, MyD88, NF-κB p65, and NF-κB p-p65 across treatment groups (*P* < 0.05, *P* < 0.01). Importantly, complementary qPCR analysis (**Figure 5B**) revealed congruent modulation at the transcriptional level, with TLR4/NF-κB mRNA expression patterns closely mirroring the Western blot protein data.

### 3.6 MHCD increases the level of miR-146a in renal tissues of IgAN rats and MCs

**Figure 6:**
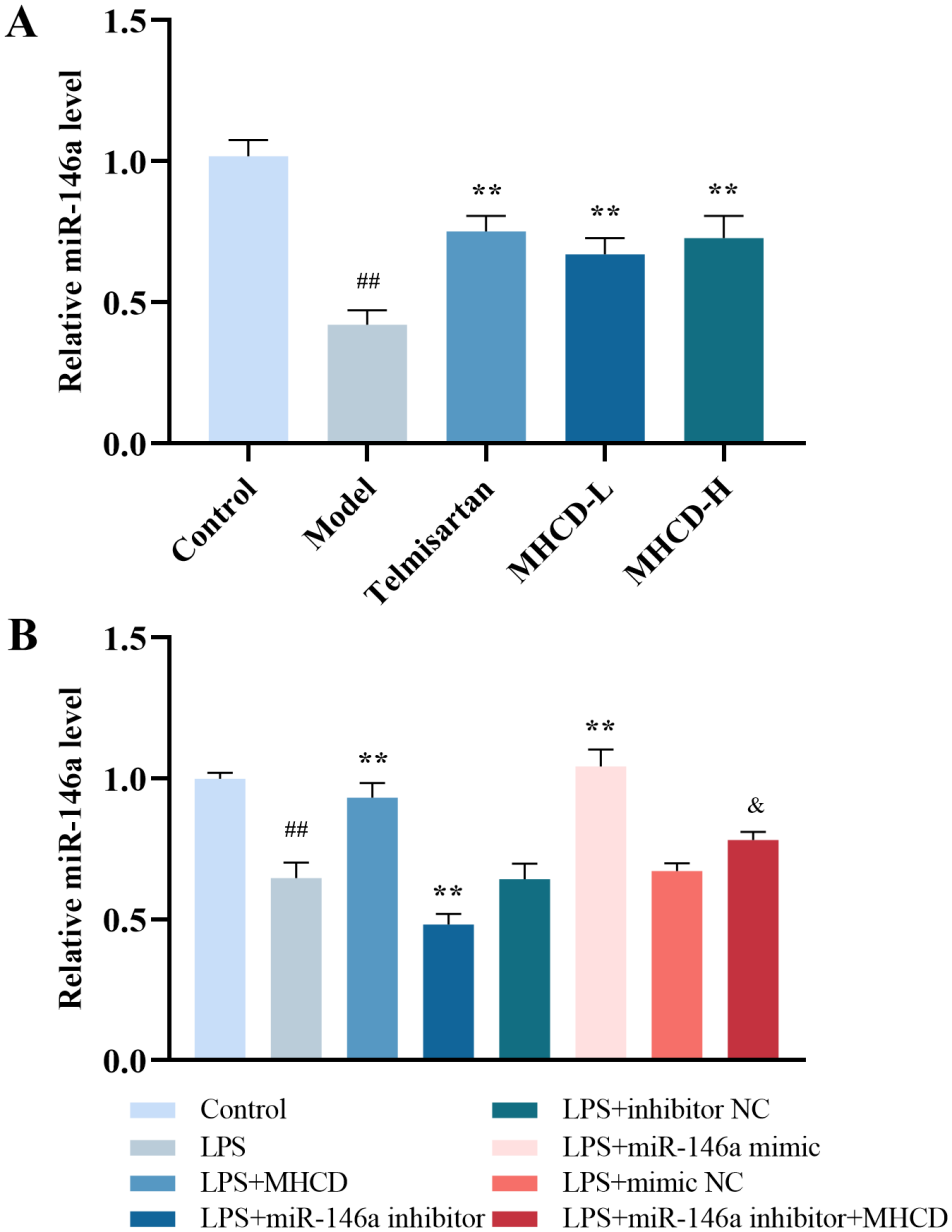
MHCD increases the level of miR-146a (*n* = 3) **(A)** Relative miR-146a level in renal tissues of IgAN rats. **(B)** Relative miR-146a level in MCs. ^##^*P <* 0.01 vs. control group; **P < 0.01 vs. model and LPS group; ^&^*P* < 0.05 vs. LPS+MHCD group.

As shown in **Figure 6A**, the expression level of miR-146a in renal tissues of IgAN rats was significantly decreased (*P* < 0.01). Both MHCD and telmisartan markedly upregulated miR-146a expression in renal tissues (*P* < 0.01). For *in vitro* validation of MHCD’s mechanism, LPS was used to induce MCs, combined with miR-146a silencing and overexpression techniques. **Figure 6B** demonstrates that LPS induction significantly reduced miR-146a expression in MCs, whereas MHCD treatment effectively restored its expression level (*P* < 0.01).

### 3.7 MHCD inhibits MCs proliferation, FN and LN expression, and inflammation

**Figure 7:**
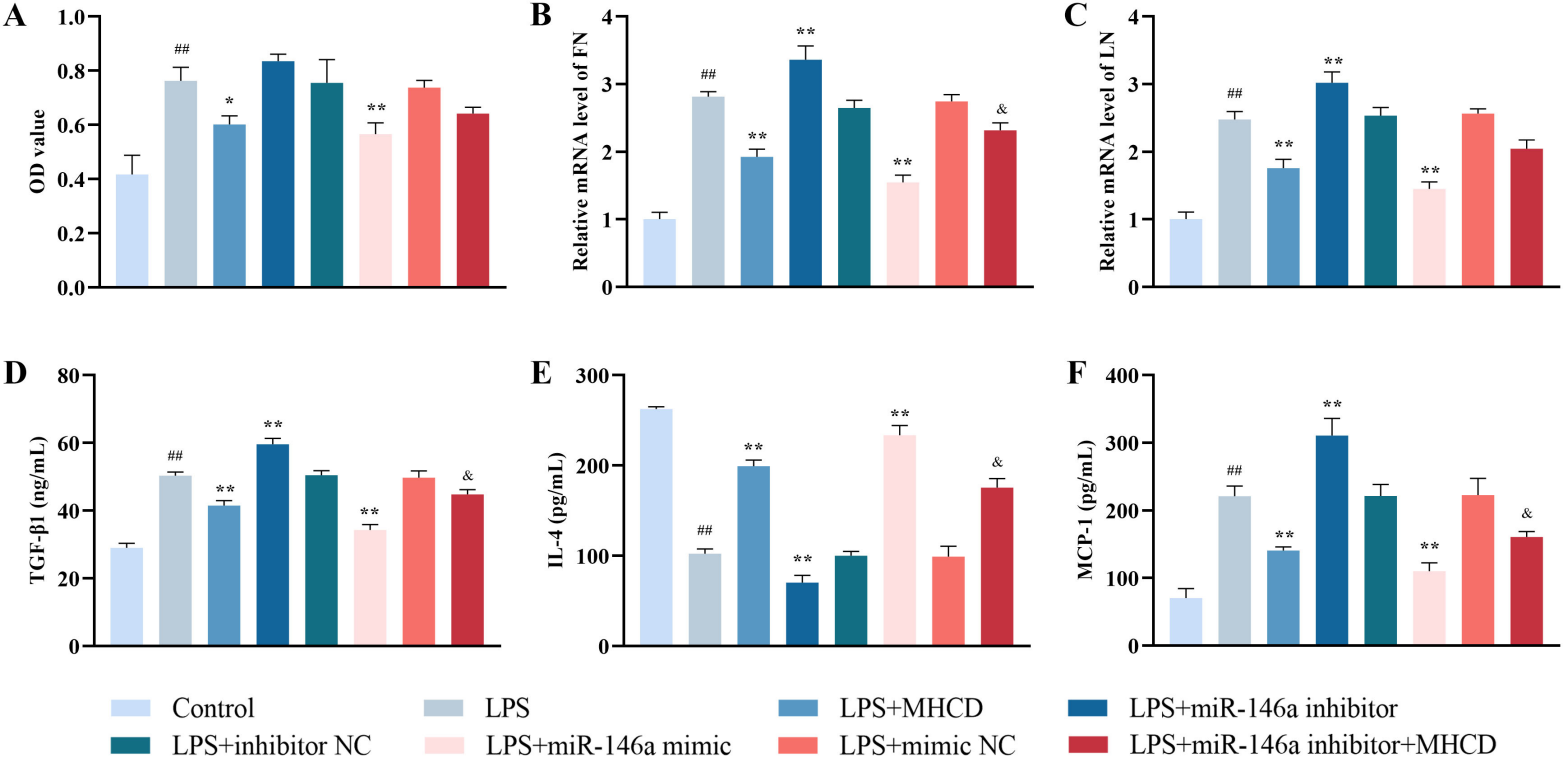
MHCD inhibits MCs proliferation, FN and LN expression, and inflammation (*n* = 3). **(A)** MCs proliferation. **(B)** Relative mRNA level of FN. **(C)** Relative mRNA level of LN. **(D)** TGF-β1 results. **(E)** IL-4 results. **(F)** MCP-1 results. ^##^*P <* 0.01 vs. control group; *P < 0.05 vs. LPS group; **P < 0.01 vs. LPS group; ^&^*P* < 0.05 vs. LPS+MHCD group.

*In vitro* experiments revealed that LPS stimulation significantly induced MCs proliferation and upregulated FN and LN expression (*P* < 0.01). The miR-146a inhibitor further exacerbated FN and LN overexpression (*P* < 0.01). MHCD effectively suppressed MCs proliferation and reduced FN and LN levels, exhibiting effects comparable to miR-146a mimic (**Figures 7A-C**). Furthermore, LPS-stimulated MCs showed elevated TGF-β1 and MCP-1 expression but decreased IL-4 levels (*P* < 0.01), a pro-inflammatory shift aggravated by miR-146a inhibition. MHCD and miR-146a mimic significantly attenuated TGF-β1 and MCP-1 secretion while restoring IL-4 production (*P* < 0.01). Notably, miR-146a inhibitor partially abolished the regulatory effects of MHCD on TGF-β1, MCP-1, and IL-4 expression (**Figures 7D-F**, *P* < 0.05).

### 3.8 MHCD upregulates miR-146a to inhibit the TLR4/NF-κB signaling pathway

**Figure 8:**
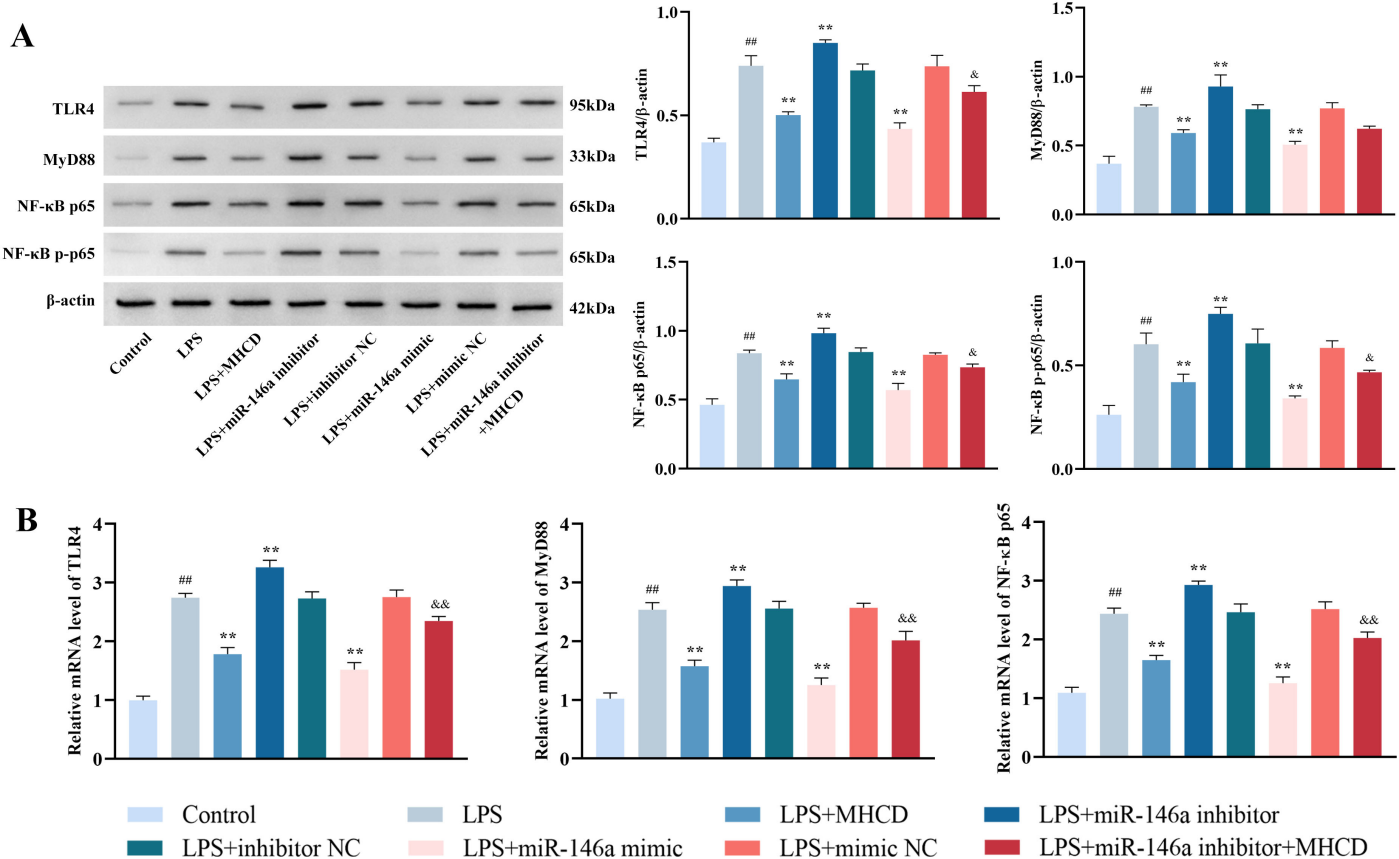
MHCD upregulates miR-146a to inhibit the TLR4/NF-κB signaling pathway (*n* = 3). **(A)** Western blot analysis of TLR4, MyD88, NF-κB p65, and NF-κB p-p65. **(B)** Relative mRNA expression of TLR4, MyD88, NF-κB p65. ^##^*P <* 0.01 vs. control group; **P < 0.01 vs. LPS group; ^&^*P* < 0.05 vs. LPS+MHCD group; ^&&^*P* < 0.01 vs. LPS+MHCD group.

Western blot analysis was performed to determine the protein levels of TLR4, MyD88, NF-κB p65, and NF-κB p-p65 in MCs (**Figure 8A**). The results demonstrated that LPS stimulation significantly upregulated the expression of TLR4, MyD88, NF-κB p65, and NF-κB p-p65 (*P* < 0.01). This LPS-induced increase was further exacerbated by miR-146a inhibitor (*P* < 0.01). MHCD and miR-146a mimic effectively suppressed TLR4, MyD88, NF-κB p65, and NF-κB p-p65 expression (*P* < 0.01). However, in the miR-146a inhibitor plus MHCD co-treatment group, the protein levels of TLR4, NF-κB p65, and NF-κB p-p65 remained significantly higher than those in the MHCD group (*P* < 0.05).

Consistent with Western blot data, qPCR analysis (**Figure 8B**) confirmed that LPS-induced increases in TLR4, MyD88, and NF-κB p65 mRNA levels were similarly modulated by miR-146a inhibitor and MHCD. Notably, miR-146a inhibition attenuated the suppressive effect of MHCD on the TLR4/NF-κB signaling pathway (*P* < 0.01), suggesting that MHCD exerts its therapeutic effects by upregulating miR-146a to inhibit TLR4/NF-κB signaling.

## 4 Discussion

IgAN is the most common primary glomerulonephritis worldwide, with nearly all patients at risk of progressing to ESKD, imposing a substantial burden on both affected families and healthcare systems (27). Most IgAN patients present with asymptomatic hematuria and proteinuria, the latter being a major risk factor for progressive renal function decline and poor prognosis. Analysis from the UK National Registry of Rare Kidney Diseases’ IgAN cohort revealed that higher proteinuria levels correlate with lower survival rates. Even in patients with time-averaged proteinuria <0.44 g/g, 20% developed ESKD or died within 10 years (27). Furthermore, a large prospective Chinese IgAN cohort study found that time-varying proteinuria >0.5 g/day significantly increased renal failure risk, particularly in those with baseline proteinuria >1.0 g/day (28).

Current IgAN treatment remains suboptimal. While supportive care—primarily renin-angiotensin system blockade—controls proteinuria and blood pressure, approximately 30% of patients show inadequate response. Immunosuppressive therapies, including corticosteroids, have demonstrated variable efficacy but carry risks of severe adverse effects. Recent advances have introduced novel therapies like Nefecon, SGLT2 inhibitors, B-cell-targeted treatments, and complement inhibitors. However, they are expensive and their long-term efficacy requires further validation (29). TCM has been applied in kidney diseases for millennia. MHCD is a clinically effective compound for managing IgAN-associated proteinuria. Our findings demonstrate that MHCD reduces proteinuria and serum Gd-IgA1 levels while ameliorating renal histopathological damage and glomerular IgA deposition, confirming its therapeutic efficacy. However, MHCD showed no significant effect on Scr or BUN. This discrepancy may be attributed to multiple factors. Urinary protein, an early sensitive marker of glomerular injury, improves earlier than Scr/BUN, and the 8-week treatment period may not have been long enough to detect statistically significant changes in mild renal impairment. Moreover, Scr and BUN are subject to extrarenal influences and exhibit considerable variability. Notably, MHCD did not impair renal function while reducing proteinuria, indicating good safety.

IgAN is an immune-mediated kidney disease whose exact pathogenesis remains incompletely understood. Currently, the internationally accepted pathogenic framework is the “multi-hit hypothesis” mediated by immunological mechanisms (1). The formation of Gd-IgA1 constitutes the initial hit, followed by recognition by circulating anti-glycan autoantibodies to form pathogenic IgA1-containing immune complexes. These complexes deposit in the glomerular mesangium, triggering the activation and proliferation of MCs and subsequent release of proinflammatory cytokines, ultimately leading to fibrotic remodeling. This cascade of inflammation and fibrosis progresses to glomerulosclerosis, tubular atrophy, and interstitial fibrosis, making the blockade of renal inflammation and fibrosis critical therapeutic targets in IgAN (30).

MCP-1, a key CC chemokine family member, recruits monocytes and macrophages to inflammatory sites, amplifying inflammatory responses. Emerging evidence implicates MCP-1 in IgAN progression and suggests its potential as a predictor of chronic kidney injury (31, 32). IL-4, a pleiotropic cytokine, exhibits context-dependent pro- and anti-inflammatory properties. While IL-4 may modestly promote aberrant IgA1 glycosylation in disease pathogenesis, corticosteroid therapy studies paradoxically show elevated serum IL-4 levels correlating with reduced proteinuria in IgAN patients (33). TGF-β1 mediates inflammatory responses while promoting mesangial matrix deposition and glomerulosclerosis (34, 35). Our findings demonstrate that MHCD significantly downregulates TGF-β1 and MCP-1 expression in both renal tissues and LPS-stimulated MCs, while upregulating IL-4 levels. Furthermore, MHCD reduces FN and LN expression in renal tissue and suppresses their mRNA transcription in MCs, concurrently inhibiting LPS-induced MCs proliferation. These results collectively indicate MHCD’s dual therapeutic actions in mitigating IgAN-associated inflammation and renal fibrosis.

The TLR4/NF-κB signaling pathway serves as a pivotal regulator of inflammatory responses. As a pattern recognition receptor, TLR4 detects danger signals like LPS and initiates MyD88-dependent signal transduction, leading to NF-κB nuclear translocation and subsequent regulation of inflammatory cytokines and chemokines (7). In renal diseases, aberrant activation of this pathway promotes MCs proliferation, podocyte injury, and tubulointerstitial fibrosis (36–38). TLRs respond to mucosal infections or abnormal antigen exposure, triggering polyclonal lymphocyte proliferation and circulating immune complex formation - key processes in Gd-IgA1 production in IgAN (39). TLR4 actively participates in IgAN pathogenesis, as evidenced by its upregulated expression in peripheral blood monocytes of IgAN patients and renal tissues of IgAN rats, showing significant correlation with proteinuria and clinical disease activity (40). These findings position the TLR4/NF-κB pathway as a crucial mechanism in IgAN progression and a promising therapeutic target. Our research observes elevated TLR4, MyD88, and NF-κB expression in renal tissues of IgAN rats and LPS-stimulated MCs, confirming their pathogenic role. Notably, MHCD significantly reduces both protein and mRNA levels of these signaling molecules across experimental models. This suppression of TLR4/NF-κB signal transduction underlies MHCD’s observed anti-inflammatory and anti-fibrotic effects in IgAN treatment.

miR-146a is a critical microRNA that plays pivotal roles in immunomodulation, inflammatory responses, and cellular proliferation. Functioning as an anti-inflammatory miRNA, it exerts therapeutic effects in various kidney diseases through suppression of the TLR4/NF-κB pathway. Clinical observations reveal a significant inverse correlation between urinary miR-146a levels and disease activity and proteinuria in lupus nephritis patients (41). Experimental studies demonstrate that miR-146a alleviates renal injury and inflammation in systemic lupus erythematosus mouse models by modulating canonical and non-canonical NF-κB signaling pathways (42). The renoprotective effects of miR-146a extend to acute kidney injury, where it attenuates TLR4/NF-κB activation and downstream transcriptional factor expression in both LPS-induced rat models and human renal proximal tubular epithelial cells (43). Upregulation of miR-146a has been shown to ameliorate microinflammation in chronic kidney disease through TLR4/NF-κB inhibition (16). Notably, miR-146a-deficient mice exhibit increased renal IgA deposition, suggesting a potential role in IgAN pathogenesis (14). However, the specific regulatory mechanism of miR-146a in IgAN through TLR4/NF-κB signaling remains incompletely understood.

Our investigation reveals that miR-146a inhibition exacerbates LPS-induced MCs proliferation, extracellular matrix (FN and LN) deposition, and inflammatory responses. Mechanistically, miR-146a silencing activates the TLR4/NF-κB axis, as evidenced by elevated levels of TLR4, MyD88, NF-κB p65, and NF-κB p-p65. Importantly, both MHCD and miR-146a mimic effectively counteract these pathological changes by restoring miR-146a expression and suppressing TLR4/NF-κB signaling. The diminished protective effects of MHCD when co-administered with miR-146a inhibitor provide compelling evidence that MHCD’s anti-inflammatory and anti-fibrotic actions in IgAN are mediated primarily through miR-146a upregulation.

An important limitation of this study is that the direct binding of miR-146a to the 3′-untranslated region of TLR4 was not verified by dual-luciferase reporter assay. Therefore, although our data support a functional correlation between miR-146a and TLR4/NF-κB pathway activity, the possibility that miR-146a indirectly modulates TLR4 signaling through other pathways, such as IRAK1/TRAF6 or STAT3, cannot be excluded. Further in−depth investigation is warranted to address these questions. In addition, the cellular experimental conditions were based primarily on our team’s previous literature and experience. Future studies should further optimize these parameters to better exclude non−specific effects and strengthen the conclusions.

## 5 Conclusion

MHCD partially attenuates renal inflammation and fibrosis in IgAN by upregulating miR-146a and thereby inhibiting the TLR4/NF-κB pathway. These findings underscore the involvement of miR-146a in IgAN pathogenesis and provide experimental evidence supporting MHCD as a potential therapeutic agent, with the miR-146a/TLR4/NF-κB axis representing a promising intervention target.

## Data Availability

The raw data supporting the conclusions of this article will be made available by the authors, without undue reservation.
